# The long‐term direct and indirect economic burden among Parkinson's disease caregivers in the United States

**DOI:** 10.1002/mds.27579

**Published:** 2018-12-27

**Authors:** Pablo Martinez‐Martin, Dendy Macaulay, Yash J. Jalundhwala, Fan Mu, Erika Ohashi, Thomas Marshall, Kavita Sail

**Affiliations:** ^1^ National Center of Epidemiology and CIBERNED Carlos III Institute of Health Madrid Spain; ^2^ Analysis Group, Inc. New York New York USA; ^3^ AbbVie Inc. North Chicago Illinois USA; ^4^ Analysis Group, Inc. Boston Massachusetts USA

**Keywords:** direct costs, income, indirect costs, Parkinson's disease, work loss

## Abstract

**Background:**

Parkinson's disease is a progressive, disabling neurodegenerative disorder associated with significant economic burden for patients and caregivers. The objective of this study was to compare the direct and indirect economic burden of Parkinson's patients’ caregivers with demographically matched controls in the United States, in the 5 years after first diagnosis of Parkinson's disease.

**Methods:**

Policyholders (18‐64 years old) linked to a Parkinson's disease patient (≥2 diagnoses of Parkinson's disease; first diagnosis is the index date) from January 1, 1998 to March 31, 2014, were selected from a private‐insurer claims database and categorized as Parkinson's caregivers. Eligible Parkinson's caregivers were matched 1:5 to policyholders with a non‐Parkinson's dependent (controls). Multivariable regression adjusted for baseline characteristics estimated direct costs (all‐cause insurer cost [medical and prescription] and comorbidity‐related medical costs; patient out‐of‐pocket costs) and indirect costs (disability and medically related absenteeism costs). Income progression was also compared between cohorts.

**Results:**

A total of 1211 eligible Parkinson's caregivers (mean age, 56 years; 54% female) were matched to 6055 controls. In adjusted analyses, Parkinson's caregivers incurred significantly higher year 1 total all‐cause insurer costs ($8999 vs $7117) and medical costs ($7081 vs $5568) (both *P* < 0.01) and higher prescription costs (range for years 1‐5, $2506‐2573 vs $1405‐$1687) and total out‐of‐pocket costs ($1259‐1585 vs $902‐$1192) in years 1‐5 (all *P* < 0.01). Parkinson's caregivers had significantly higher adjusted indirect costs in years 1‐3 (range for years 1‐3, $2054‐$2464 vs $1681‐$1857; all *P* < 0.05) and higher cumulative income loss over 5 years ($5967 vs $2634 by year 5; *P* for interaction = 0.03).

**Conclusions:**

Parkinson's caregivers exhibited higher direct and indirect costs and greater income loss compared with matched controls. © 2018 International Parkinson and Movement Disorder Society © 2018 The Authors. *Movement Disorders* published by Wiley Periodicals, Inc. on behalf of International Parkinson and Movement Disorder Society.

Parkinson's disease (PD) is a chronic, progressive neurodegenerative disease with physical symptoms manifesting as tremor at rest, rigidity, bradykinesia, and postural instability.^1^, ^2^, ^3^ PD is the second most prevalent neurodegenerative disease after Alzheimer's disease, affecting approximately 4‐10 million people worldwide, and is expected to double in prevalence by 2030 as the population ages.^1^, ^4^ In the United States, the prevalence of PD was estimated at 630,000 in 2010, with higher incidence among white men compared with other groups.^5^, ^6^, ^7^ Age at onset of PD is typically around age 60 but can start earlier.^8^ In addition to motor symptoms, cognitive deterioration, neuropsychiatric disturbances (eg, depression, apathy, anxiety, and hallucinations), and behavioral disturbances (eg, impulse control disorders) are common among PD patients.^1^, ^9^ There is currently no cure for PD, and although treatment options such as dopamine replacement (ie, levodopa) or agonists can help to alleviate symptoms, none slow the progression of the disease.

PD may present a substantial burden to the caregivers of PD patients. PD patients may need help with many aspects of life, including personal safety, mobility, transportation, medication compliance, daily activities, and social involvements.^10^, ^11^ These challenges, along with the behavioral disturbances of PD patients and the long duration of the disease, can be very difficult for PD caregivers to manage, which may impact their health outcomes and as a result increase health‐care spending.^1^, ^10^, ^11^, ^12^, ^13^, ^14^, ^15^, ^16^ In addition to the physical and mental tolls of caretaking, caregivers of PD patients face a significant economic burden because of the costs of caring, decreased work productivity, and reduced wages. These indirect costs are substantial among caregivers, and approximately 30%‐40% of the total indirect costs associated with PD are attributable to lost earnings by caregivers and patients as well as hours required for caregiving.^10^ More than 25% of caregivers spend more than 70 hours per week caring for PD patients.^11^ Furthermore, a reported 26% of PD caretakers had to reduce or give up work, and 30.4% reported that their finances worsened as a result of caregiving.^10^

There is a lack of literature comprehensively assessing the burden of PD on PD caregivers. Publications on PD caregivers’ burden have largely focused on the noneconomic aspects of burden or the impact on quality of life (QoL). In addition, to date, no studies have assessed the lost earnings of PD caregivers over time. The indirect cost of caregiving has been primarily estimated as the monetary value of work loss associated with caregiving rather than actual earnings lost.^17^, ^18^ Moreover, no studies have evaluated how the burden of PD caregivers changes over time; however, because the burden of caring may increase as PD progresses,^19^ there is a need for long‐term studies assessing the PD caregivers’ burden over time. To address this literature gap, the current study aimed to compare the direct health‐care burden (from both health‐care insurer and patient perspectives), indirect costs (including disability and medically related absenteeism costs), and income progression (ie, how income changed over time) between caregivers of newly diagnosed PD patients and matched controls during a 5‐year period in the US working‐age population.

## Methods

### Data Source

Data for this study, spanning January 1, 1998, to March 31, 2014, were obtained from OptumHealth Care Solutions, Inc., a deidentified private‐insurer claims database. This database contains administrative claims for >18 million privately insured individuals covered by 84 self‐insured Fortune 500 companies with locations across the United States. Medical and drug claims as well as eligibility data are available for all beneficiaries (ie, employees, spouses, dependents, and retirees), whereas measures of work loss (ie short‐ and long‐term disability claims) were available for primary policyholders (ie, employees) in 43 companies. Employer‐reported income data were available for a subgroup of employees. Data were anonymized and compliant with the requirements of the Health Insurance Portability and Accountability Act; thus, no institutional board review was required. This study was conducted in accordance with the Declaration of Helsinki.

### Study Design

#### *Sample Selection and Cohort Construction*

The study consisted of 2 cohorts: (1) PD caregivers and (2) matched controls who were not PD caregivers. For the PD caregiver cohort, the index date was the date of the PD patient's first PD diagnosis. For the control cohort, the index date was randomly assigned. PD patients were identified as having ≥2 diagnosis for PD (International Classification of Diseases, Ninth Revision–Clinical Modification [ICD‐9‐CM] code 332.0). Subjects were eligible for inclusion in the PD caregiver cohort if they were primary policyholders linked to a nonprimary policyholder PD patient, had continuous eligibility of at least 6 months before (baseline period) and at least 12 months following the index date, and were aged 18‐64 years during the baseline and the study periods (see definitions below). Controls were selected from primary policyholders if they did not have a family member with PD, had continuous eligibility of at least 6 months before (baseline period) and at least 12 months following the index date, and were aged 18‐64 during the baseline and study periods. Cases were matched 1:5 to controls who were randomly selected from the control pool that satisfied the requirement described above.

For the direct cost analysis, all PD caregivers and controls meeting the above criteria were included. For the indirect cost analysis, PD caregivers and their controls were also required to be employed and have work loss data during the baseline and 12 months post–index date. For the income progression analysis, PD caregivers and their controls were also required to be employed and have income data during the calendar year of the index date (index calendar year) and for at least 1 calendar year of follow‐up. For each of the analysis, controls were randomly matched to PD caregivers in a 5:1 ratio exactly on sex, age, region, and index year.

In addition, PD patients linked to the PD caregivers and the oldest dependent of each of the matched control patients were selected if they had at least 6 months of continuous eligibility prior to the index date (baseline period).

#### *Study Periods*

The study period was defined as the period of time following the index date until the earlier of the following: (1) the patient turned 65 years old or (2) the patient lost continuous eligibility. Each patient's study period was at least 1 year (per sample selection) and was capped at 5 years. For the indirect cost and income progression analyses, patients were also required to have continuous employment during the study period.

### Baseline Characteristics

Information collected for the PD caregivers and matched controls during the baseline period included demographic characteristics (ie, age, sex, and region), index year, insurance type, the Charlson Comorbidity Index (CCI), and caregiving‐related comorbidities identified in previous studies.^20^, ^21^, ^22^, ^23^, ^24^ Caregiving‐related comorbidities were identified based on a targeted review of literature on the caregiving burden in other diseases (eg, rheumatoid arthritis, osteoarthritis, back pain, and migraine). In addition, the baseline characteristics of the selected PD patients and dependents of the matched controls were also summarized.

### Outcomes

#### *Direct Costs*

Direct insurer and out‐of‐pocket costs were estimated during each year in the study period. Direct insurer costs were based on reimbursements from third‐party payers to health‐care providers and pharmacies. Direct insurer cost outcomes included all‐cause medical costs, prescription drug costs, total direct costs (medical and prescription drug costs), and comorbidity‐related medical costs (calculated as the sum of medical costs associated with an ICD‐9 code for a caregiving‐related comorbidity). Direct out‐of‐pocket costs were based on payments from beneficiaries to health‐care providers and pharmacies and were calculated as the sum of deductible, coinsurance, and copayment amounts. Out‐of‐pocket cost outcomes included all‐cause medical, prescription drug, and total out‐of‐pocket costs.

#### *Indirect Costs*

Indirect costs among patients with work loss data and were estimated during each year in the study period. Costs were estimated based on medically related absenteeism costs (calculated based on medically related absenteeism days and employee income) and disability costs (based on actual employer disability payments from disability claims).

#### *Income Progression*

Income progression, or how income changed over time, among patients with income data was estimated during the index calendar year and each calendar year in the follow‐up period following the index calendar year. Income included wages and salary, excluding variable components such as equity options.

### Statistical Analyses

Baseline characteristics were described using means and standard deviations (SDs) for continuous variables and frequencies and percentages for categorical variables. Characteristics were compared between PD caregivers and controls using generalized estimating equations (GEEs) to account for matching.

Direct and indirect costs were estimated and compared between PD caregivers and controls during each year of the study period, both descriptively and following adjustment for baseline characteristics (age, sex, region, health plan, CCI, and index year). In the descriptive analysis, mean costs were compared using GEEs to account for matching. In the adjusted analysis, longitudinal generalized linear mixed models with a Tweedie distribution and a log link function were used to account for the correlation between the PD caregivers and their matched controls as well as the correlation within patients because of the repeated measures of costs. Bootstrapped 95% confidence intervals (CIs) and 2‐sided *P* values were computed for mean differences in costs between PD caregivers and controls.

Annualized income was estimated and compared between PD caregivers and controls during each calendar year of the study period. Income progression was assessed using a longitudinal generalized linear mixed model with a gamma distribution and log link. The model adjusted for baseline characteristics including age, sex, region, health plan, CCI, and index year. Both costs and income were inflated to 2014 US dollars using the Consumer Price Index from the US Bureau of Labor Statistics.

All analyses were conducted in SAS 9.3. A *P* < 0.05 was used to determine significance during comparisons.

## Results

### Sample Selection and Cohort Construction

**Figure 1:**
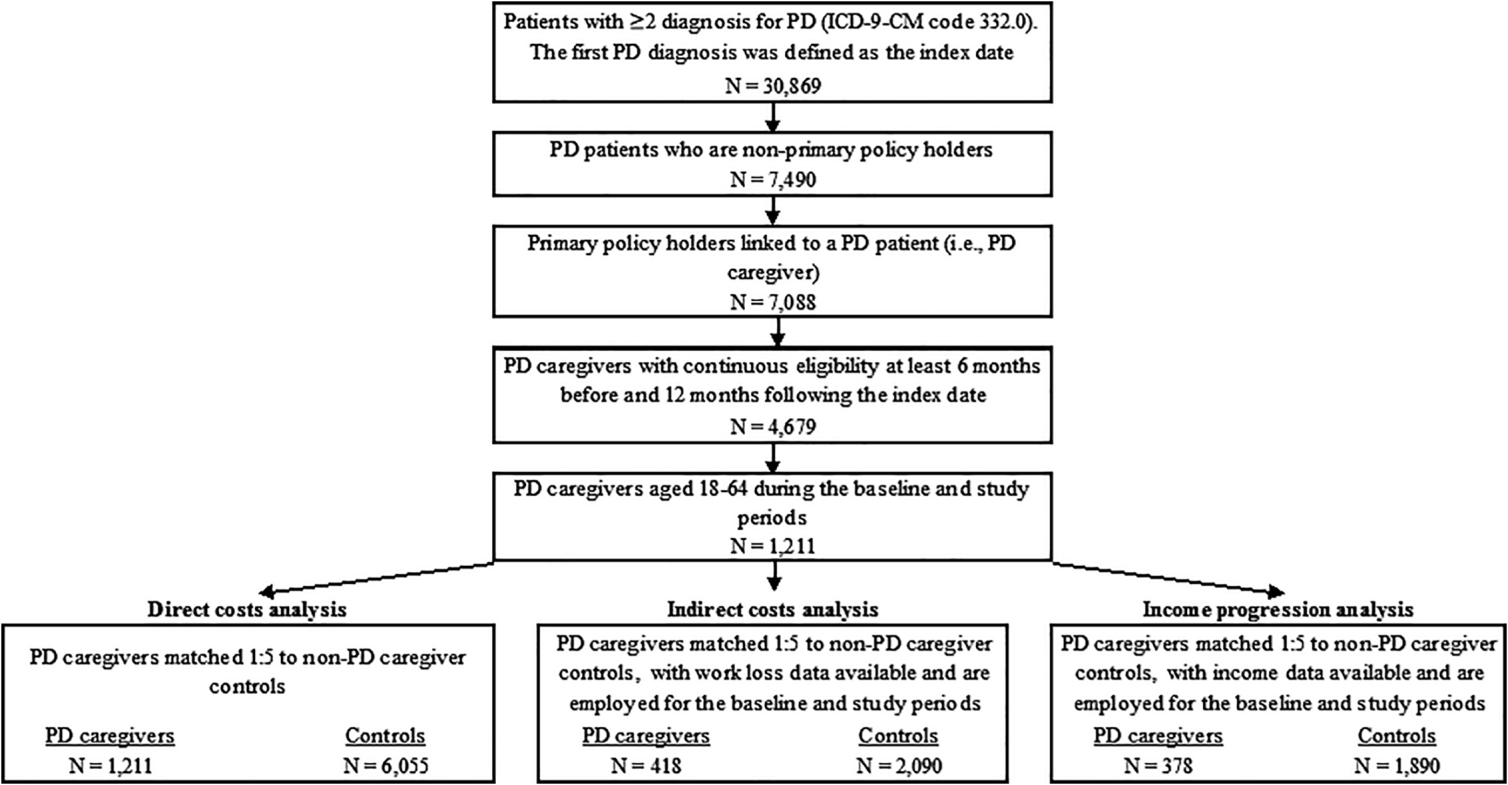
Sample selection. ICD‐9‐CM, International Classification of Diseases, Ninth Revision, Clinical Modification; N, number; PD, Parkinson's disease.

The sample selection for PD and control caregivers included in the 3 analyses (direct costs, indirect costs, and income progression) is illustrated in Figure 1. A total of 1211 PD caregivers were included in the direct cost analysis and were matched to 6055 control caregivers. Of these PD caregivers, 418 employees with work loss data were included in the indirect cost analysis and were matched to 2090 controls. Among the 1211 PD caregivers included in direct cost analysis, 378 employed PD caregivers with income data were included in the income progression analysis and were matched to 1890 controls. The sample sizes for each analysis and for each year during the study period are described in Supplemental Table 1. The loss to follow‐up rate ranged from 50% to 65% in year 3 and from 73% to 90% in year 5.

### Baseline Characteristics

For the PD caregivers and matched controls in the direct cost analysis, the mean age was 55.7 years, and 54.2% were female (Table 1). PD caregivers had significantly higher rates of several general caregiving‐related comorbidities (backache, hypertension, gastroesophageal reflux disease [GERD]/heartburn, and irritable bowel syndrome; all *P* < 0.05) compared with matched controls (Table 1). Other caregiving‐related comorbidities (eg, anxiety, depression, etc.) were comparable between PD caregivers and matched controls. The characteristics for patients in the indirect and income progression analyses were comparable to those for patients in the direct cost analysis (not shown; available on request).

**Table 1:** Baseline characteristics of PD caregivers and matched controls^a^

	PD caregivers		Matched controls		*P* f
	(n = 1211)		(n = 6055)		
Demographic characteristics^b^					
Sex, n (%)					—
Female	656	(54.2%)	3280	(54.2%)	
Age (years), mean (SD)	55.73	(6.13)	55.73	(6.13)	—
Age distribution, n (%)					—
18‐30 years	1	(0.1%)	5	(0.1%)	
31‐44 years	70	(5.8%)	350	(5.8%)	
45‐54 years	347	(28.7%)	1735	(28.7%)	
55‐64 years	793	(65.5%)	3965	(65.5%)	
Year of index date, n (%)					—
1998‐2001	122	(10.1%)	610	(10.1%)	
2002‐2005	322	(26.6%)	1610	(26.6%)	
2006‐2009	359	(29.6%)	1795	(29.6%)	
2010‐2013	408	(33.7%)	2040	(33.7%)	
Region, n (%)^c^					—
Northeast	262	(21.6%)	1310	(21.6%)	
Midwest	299	(24.7%)	1495	(24.7%)	
South	463	(38.2%)	2315	(38.2%)	
West	187	(15.4%)	935	(15.4%)	
Insurance type					
Preferred provider organization	721	(59.5%)	3583	(59.2%)	0.613
Point of service	291	(24.0%)	1425	(23.5%)	
Indemnity	151	(12.5%)	748	(12.4%)	
Other	48	(4.0%)	299	(4.9%)	
Comorbidities, n (%)^d^					
Select comorbidities					
Anxiety	36	(3.0%)	128	(2.1%)	0.070
Depression	56	(4.6%)	244	(4.0%)	0.351
Sleep problem	18	(1.5%)	64	(1.1%)	0.208
Backache	200	(16.5%)	755	(12.5%)	< 0.001^g^
Hypertension	297	(24.5%)	1231	(20.3%)	0.001^g^
GERD/heartburn	55	(4.5%)	203	(3.4%)	0.046^g^
Irritable bowel syndrome	18	(1.5%)	36	(0.6%)	0.001^g^
Angina	13	(1.1%)	53	(0.9%)	0.498
Migraine/headache	39	(3.2%)	178	(2.9%)	0.607
Hearing loss	14	(1.2%)	50	(0.8%)	0.261
Arthritis‐related condition	200	(16.5%)	939	(15.5%)	0.373
Cataract	32	(2.6%)	148	(2.4%)	0.686
Charlson Comorbidity Index, mean (SD)^e^	0.31	(0.75)	0.30	(0.82)	0.614

GERD, gastroesophageal reflux disease; ICD‐9, International Classification of Diseases, Ninth Revision; n, number; PD, Parkinson's disease; SD, standard deviation.

a The baseline period was defined as the 6 months prior to the index date.

b The PD caregivers and matched controls were matched on sex, age in years, index year, and region.

c The distribution of region is reflective of the regional distribution of claims data available and not the regional distribution of PD caregivers.

d Comorbidities were defined using ICD‐9 codes during the baseline period.

e The 17 conditions included in the Charlson Comorbidity Index were identified using ICD‐9 diagnosis codes reported by Romano et al^25^ during the baseline period.

f *P* values were estimated by generalized estimating equations, which account for the correlation between the PD caregivers and their matched controls.

g Statistically signicant at *P* < 0.05.

The demographic characteristics of the PD patients (n = 1146) and control dependents (n = 3063) are shown in Supplemental Table 2. The vast majority of the PD patients and control dependents were spouses of the primary policyholders, although the proportion was higher for PD patients (98.2% vs 91.1%, respectively; *P* < 0.01). The PD patients were older compared with the control dependent cohort (58.6 vs 51.2 years, respectively; *P* < 0.01) and had a significantly higher mean ± SD CCI score (0.74 ± 1.34 versus 0.24 ± 0.74; *P* < 0.01).

### Direct Insurer Costs

In the adjusted analysis, PD caregivers had significantly higher mean total all‐cause costs ($8999 vs $7117, respectively), mean all‐cause medical costs ($7081 vs $5568), and mean comorbidity‐related medical costs ($1589 vs $1228) compared with matched controls in the first year of the study period (all *P* < 0.01), but comparable costs in years 2‐5 (Table 2). Prescription drug costs were largely stable for both cohorts over all 5 years; however, PD caregivers had significantly higher mean adjusted prescription drug costs compared with controls in all 5 years (range for years 1‐5, $2506‐$2573 vs $1405‐$1687, respectively; all *P* < 0.01; Table 2).

**Table 2:** Adjusted prescription and total direct insurer costs and out‐of‐pocket costs during the 5‐year study period^a^

	Total insurer costs^b^^,^ c			Prescription drug, insurer costs^b^^,^ c			Total OOP costs^d^^,^ e			Prescription OOP costs^d^^,^ e		
	PD caregivers	Matched controls	*P* ^6^	PD caregivers	Matched controls	*P* ^6^	PD caregivers	Matched controls	*P* ^6^	PD caregivers	Matched controls	*P* f
Year 1	$8999 ($8022‐$10,138)	$7117 ($6574‐$7650)	< 0.01^g^	$2506 ($2180‐$2906)	$1430 ($1345‐$1517)	< 0.01^g^	$1259 ($1168‐$1355)	$902 ($868 ‐ $934)	<0.01^g^	$494 ($445 ‐ $553)	$317 ($304 ‐ $331)	< 0.01^g^
Year 2	$8265 ($7226‐$9356)	$7453 ($6757‐$8077)	0.12	$2489 ($2161‐$2896)	$1405 ($1308‐$1498)	< 0.01^g^	$1292 ($1189‐$1398)	$977 ($936‐$1016)	<0.01^g^	$488 ($437 ‐ $546)	$314 ($300 ‐ $330)	< 0.01^g^
Year 3	$8880 ($7430‐$10,298)	$8190 ($7425‐$9039)	0.42	$2457 ($2104‐$2834)	$1486 ($1378‐$1589)	< 0.01^g^	$1329 ($1210‐$1456)	$1077 ($1023‐$1137)	<0.01^g^	$489 ($432 ‐ $549)	$331 ($314 ‐ $349)	< 0.01^g^
Year 4	$8370 ($6994‐$9986)	$9038 ($7771‐$10,233)	0.46	$2362 ($1974‐$2800)	$1512 ($1395‐$1636)	< 0.0^g^	$1434 ($1290‐$1587)	$1144 ($1082‐$1212)	<0.01^g^	$495 ($431 ‐ $566)	$341 ($320 ‐ $363)	< 0.01^g^
Year 5	$8774 ($7137‐$10,774)	$8572 ($7395‐$9837)	0.77	$2573 ($2121‐$3065)	$1687 ($1532‐$1853)	< 0.01^g^	$1585 ($1416‐$1807)	$1192 ($1,108‐$1273)	<0.01^g^	$551 ($467 ‐ $634)	$370 ($344 ‐ $400)	< 0.01^g^

Costs are listed as means (95% confidence intervals).

CI, confidence interval; OOP, out of pocket; PD, Parkinson's disease.

a The study period for each patient was index date to the end of eligibility (maximum follow‐up of 5 years) or until the patient was age 65.

b Total insurer costs included medical and drug costs and were calculated as the sum of paid amounts. Costs were adjusted for inflation to 2014 US dollars using the Consumer Price Index from the Bureau of Labor Statistics.

c Insurer costs were adjusted for age, index year, region, sex, Charlson Comorbidity Index, and health‐care plan.

d Total out‐of‐pocket costs included medical and drug costs and were calculated as the sum of deductible, coinsurance, and copayment amounts. Costs were adjusted for inflation to 2014 US dollars using the Consumer Price Index from the Bureau of Labor Statistics.

e Out‐of‐pocket costs were adjusted for age, index year, region, sex, Charlson Comorbidity Index, and health‐care plan.

f *P* values were 2 sided and were computed based on the distribution of bootstrapped mean differences in costs between PD caregivers and controls.

g Statistically significant at *P* < 0.05.

### Direct Out‐of‐Pocket Costs

In all 5 years, PD caregivers had significantly higher mean adjusted total out‐of‐pocket costs compared with controls (range years 1‐5, $1259‐1585 vs $902‐$1192, respectively; all *P* < 0.01; Table 3). In addition, PD caregivers had significantly higher mean adjusted out‐of‐pocket prescription costs ($488‐$551 vs $314‐$370, respectively; all *P* < 0.01) and medical costs ($880‐$1214 vs $561‐$826; all *P* < 0.01) compared with controls in all years.

**Table 3:** Adjusted indirect costs during the five‐year study period^a^

Indirect costs^b^	Total indirect costs,^c^ mean (95% CI)		
	PD caregivers	Matched controls	*P* d
Year 1	$2054 ($1778‐$2412)	$1709 ($1526‐$1912)	0.02^d^
Year 2	$2148 ($1804‐$2545)	$1691 ($1499‐$1908)	0.01^d^
Year 3	$2464 ($1924‐$3307)	$1857 ($1602‐$2176)	0.03^d^
Year 4	$1631 ($1330‐$1997)	$2089 ($1654‐$2683)	0.06
Year 5	$1935 ($1462‐$2591)	$2145 ($1695‐$2727)	0.60

CI. confidence interval; PD. Parkinson's disease.

a The study period for each patient was index date to the earliest date of end of eligibility, end of continuous employment, or until the patient was age 65 (maximum follow‐up of 5 years).

b All costs were inflated to 2014 US dollars using average hourly compensation data from the Bureau of Labor Statistics.

c Total indirect costs were calculated as the sum of disability and medically related absenteeism costs. Medically related absenteeism costs were calculated based on individual employee wage information obtained from eligibility files and days of medically related absenteeism. Medically related absenteeism days were imputed based on use of medical services during business days (eg, an office visit or a hospital inpatient visit Monday through Friday) as well as the waiting period in advance of the start of disability (eg, 5 missed days of work because of illness). The methodology assumed that each hospitalization day and emergency department visit accounted for a full day of work loss, whereas each outpatient/other visit accounted for half a day of work loss.

*P* values were 2‐sided and were computed based on the distribution of bootstrapped mean differences in costs between PD caregivers and controls.

d Significant difference at *P* < 0.05.

### Indirect Costs

PD caregivers had significantly higher total mean adjusted indirect costs in the first 3 years of the study period compared with controls (year 1, $2054 vs $1709; year 2, $2148 vs $1681; year 3, $2464 vs $1857, respectively; all *P* < 0.05), and costs were comparable for years 4 and 5 (Table 3).

The descriptive results for direct insurer costs, direct out‐of‐pocket costs, and indirect costs are presented in Supplemental Tables 3‐5.

### Income Loss Progression

**Figure 2:**
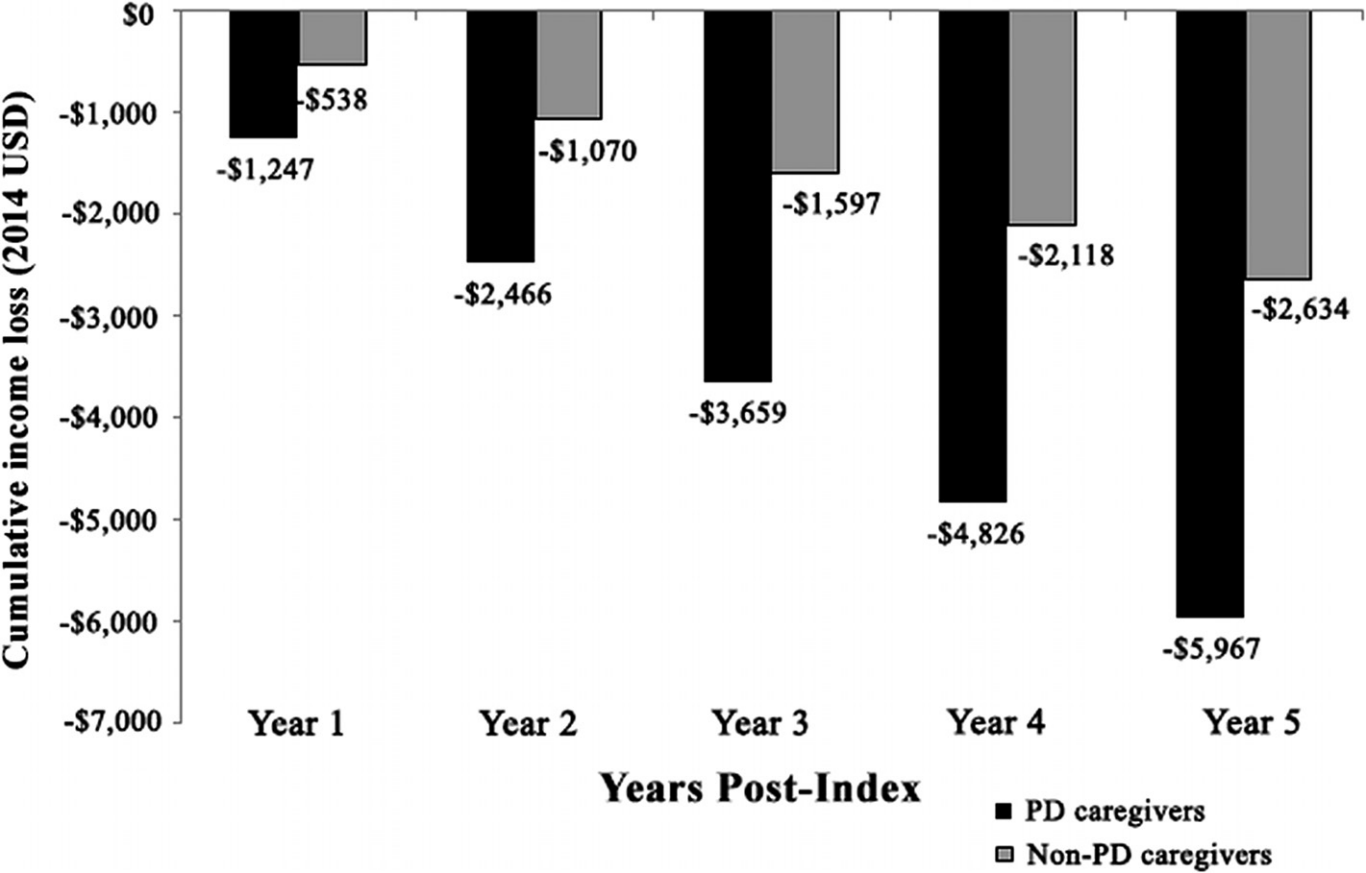
Regression‐adjusted cumulative income loss for PD caregivers versus matched controls.^a‐d^ PD, Parkinson's disease; USD, United States dollars. ^a^The study period for each patient was index date to the earliest date of end of eligibility, end of continuous employment, or until the patient was age 65 (maximum follow‐up of 5 years). ^b^Employer‐reported annualized income (including wage and salary, and excluding variable components such as equity options) available in the eligibility records for each patient were used. Employees’ income for each calendar year of observation was calculated as the average of the annualized employer‐reported income reported at each month of the corresponding calendar year in the database. Income was adjusted for inflation to 2014 USD using the Consumer Price Index from the Bureau of Labor Statistics. ^c^The regression model used was a generalized linear mixed model with a gamma distribution and log link, which accounted for the correlation in income between the PD caregivers and their matched controls as well as the correlation within patients because of the repeated measures of income. ^d^The regression‐adjusted annual income presented here represents geometric means to account for the skewness of income distribution. These were calculated by taking the average of the ln (annual income) across patients, then exponentiating this average.

The regression‐adjusted cumulative income loss for PD caregivers compared with controls is displayed in Figure 2. The annual incomes of PD caregivers and controls in the index calendar year were similar ($69,630 vs $66,425, respectively). The annual rate of income loss in the PD caregivers was more than twice the income loss in the controls (approximately $1200 vs $520 annually). Similarly, PD caregivers had more than 2 times the cumulative income loss during the 5 calendar years following the index date compared with controls ($5967 vs $2634 by year 5; *P* for interaction = 0.03).

## Discussion

This study estimated the 5‐year economic burden of caregivers of newly diagnosed PD patients in terms of direct costs, indirect costs, and cumulative income loss to assess the comprehensive burden of PD caregivers over time. The results of this study indicate that caregivers of newly diagnosed PD patients incurred higher total direct health‐care burden (total insurer and total out‐of‐pocket costs) and total indirect costs than controls during the first year after PD diagnosis. In addition, many direct cost components (insurer prescription drug costs and out‐of‐pocket medical and prescription drug costs) were significantly higher among PD caregivers than controls over all 5 years, and total indirect costs were significantly higher among PD caregivers during the first 3 years of the study period. Last, greater cumulative income loss was observed among PD caregivers compared with controls over the 5 years of the study period.

Compared with control caregivers, PD caregivers displayed higher rates of several general caregiving comorbidities at baseline (ie, backache, hypertension, GERD/heartburn, and irritable bowel syndrome), as well as numerically higher caregiving‐related comorbidity costs in all years of the study period. These results are consistent with the finding from a case‐control study of early‐onset PD in Ireland, which found that PD caregivers were observed to have an increase in chronic illness compared with noncaretaking spouses.^26^ The increased comorbidity burden among PD caregivers in the current study was observed before PD patients’ first observed PD diagnosis, which may be because of the stress caregivers were experiencing during the diagnosis phase and/or delayed PD diagnosis. This early manifestation of a negative health impact could also be explained by prior studies, which reported that PD patients’ nonmotor symptoms may be present even decades before motor symptoms manifest and possibly prior to PD diagnosis.^27^, ^28^, ^29^ We speculate that such incremental comorbidity burden may be related to increased mental and physical stress; however, the causes and pathways of such physical burden need to be further explored.

The present study results indicate that both direct and indirect cost contributors drive the higher economic burden among caregivers of PD patients compared with controls. In terms of direct costs, the finding that out‐of‐pocket medical costs, but not insurer medical and total costs, were significantly higher among PD caregivers compared with controls during all 5 years of the study period may be explained by 2 reasons. First, there was large variation in insurer costs across patients. Second, unlike insurer costs, out‐of‐pocket costs are more closely related to the frequency of health resource utilization (HRU), and therefore higher out‐of‐pocket costs may be a direct reflection of the increased HRU among PD caregivers. In terms of indirect costs, PD caregivers experienced significantly higher total indirect costs in the first 3 years of the study period, but comparable indirect costs during the last 2 years of the study period. The total indirect costs consisted of up to approximately 35% of disability costs and 65% of medical‐related absenteeism costs. The fluctuation of total indirect cost results over the study period might be because of the small sample size in the later years of the study period (eg, a small number of PD caregivers with disability in years 4‐5) and uneven attrition between the 2 cohorts.

Both the PD caregivers and matched controls experienced a loss of income during the study period, although PD caregivers experienced twice the income loss of controls based on actual income reported by the employers ($5967 vs $2634 cumulatively by year 5, respectively). The universal loss of income by both cohorts could be because of a trend of general wage stagnation in the United States during the years assessed (January 1, 1998, to March 31, 2014)^30^ or transitioning to a job with fewer hours or lower responsibility (ie, less pay) as a consequence of increased time dedicated to caregiving.

There is a lack of literature on the direct and indirect costs of caring for PD patients, as prior studies have mainly focused on the noneconomic burden among PD caregivers or the impact on QoL. According to a review of the PD caregiver literature conducted in 2014, the financial strain for caregivers was assessed in terms of missed work days and qualitative reporting of financial issues; other previously studied aspects of caregiver burden have included physical (daily activities and mobility) and psychosocial (anxiety, depression, impulse control, and isolation) functions.^31^ A study by Kowal et al estimated care recipient and PD caregivers’ reduced ability to participate in the labor market; on average, the annual household income of PD patients and their spousal caregivers was reduced by $7730 after controlling for demographic characteristics.^5^ However, the present study quantifies the income loss of the caregiver specifically, providing a new perspective on income progression and cumulative income loss among PD caregivers.

Because of the high costs associated with PD‐related care,^5^ the increasing incidence and prevalence of PD^32^ and the aging population of the United States overall,^33^ it is important to understand the economic burden on PD caregivers.^34^ In addition, direct costs may be even higher among elderly caretakers given their own predisposition to higher need for health care and higher physical strain that could be imposed.^35^ However, older caregivers are more likely to be retired or unemployed, thus the results of this study regarding the indirect costs of PD caregiving are not likely to generalize to elderly caregivers.^36^ The high prevalence of PD in the United States further underscores the magnitude of the burden of this disease. Although there are no estimates on the number of caregivers for patients with PD in the United States, the total number of patients with PD (currently estimated to be between 700,000 and 900,000 Americans^37^) presents a likely scenario that many thousands of caregivers may be impacted by the present findings. Future studies of the income loss and direct and indirect economic burdens associated with PD caretaking are warranted to understand the impact on this vulnerable population.

A strength of this study is the 5‐year time horizon for cost analysis of PD caregivers. The economic burden associated with caregiving was already evident among newly diagnosed PD patients, which provides new insights into the caregiver burden and highlights the incremental burden of the caregivers in the early phase of PD patients’ disease journey. However, PD caregiver costs in this study may be lower than those incurred by caregivers of an average PD patient because PD severity has been identified as a determinant of a caregiver's overall burden and PD caregiver costs are likely to increase as PD advances.^31^ Among patients with advanced stages of PD, debilitating physical and neuropsychological problems can increase the complexity of care. As PD progresses and oral medication such as levodopa becomes less effective, the independence of PD patients declines and patients often require full‐time complex care that increases the burden on PD caregivers.^1^, ^19^, ^38^, ^39^ Thus, future research could focus on the progression of the economic and health burden (both physical and mental) of PD on caregivers over a longer period.

### Limitations

This study should be considered in light of several limitations, some of which are inherent to claims database analyses. An important limitation of this study is the high loss‐to‐follow‐up rate over time and the lack of longitudinal follow‐up (eg, over a 10‐year time frame compared with 5 years in the current study). Although this is similar to other insurance claims databases, this high loss‐to‐follow‐up rate may have influenced the present findings in an unpredictable way. For example, it is possible that the lack of a linear trend for direct and indirect costs over time was partially because of the loss to follow‐up of highly burdened disabled PD caregivers, resulting in an underestimation the burden among the PD caregiver cohort. In addition, because of the high loss‐to‐follow‐up rate, the study was not able to evaluate PD caregivers’ incremental economic burden over a longer time frame such as 10 years. PD is a progressive disease, although the progression rate varies across patients, so future studies using databases with longer follow‐up times are warranted to demonstrate how the burden of caregiving progresses over periods greater than 5 years.

Another limitation of the study is that because PD caregivers were required to be working adults who were privately insured primary policyholders and under the age of 65, the study results may not reflect the economic burden among all PD caregivers in the United States. Although if the elderly nonworking population were studied, the indirect cost could not be assessed. Thus, future studies should assess the direct costs and comorbidities associated with caring for PD patients if focusing on an elderly population. It is possible that the incremental burden is greater in this more vulnerable population. However, it is also possible that the older caregivers are less likely to bear the full caregiving burden (their older PD spouse may be institutionalized), resulting in a lesser incremental burden compared with the working‐age and under age 65 study population of this study.

In addition, it was also assumed that the primary policyholder linked to a PD patient was the primary caregiver. Thus, if PD patients were primarily taken care of by other family members, PD caregivers’ economic burden may have been underestimated in this study. Finally, PD patients may have also received professional care not covered by insurance or could have been institutionalized during the course of the study.

## Conclusion

This is the first study to provide comprehensive information on the 5‐year economic burden of PD on working‐age caregivers. The current results suggest that the economic burden associated with caregiving is already evident among caregivers of newly diagnosed PD patients and remains high over the 5 years after PD diagnosis.

## Previous presentation

A synopsis of the current research was presented in poster format at the 19th International Congress of Parkinson's Disease and Movement Disorders meeting, which took place in San Diego, California, June 14‐June 18, 2015.

## Specific author contributions

All authors (*T. Marshall, Y*. Jalundhwala, K. Sail, *D. Macaulay, F. Mu, E. Ohashi, and P*. Martinez‐Martin) participated in the design of the study and contributed to the manuscript development. Data were analyzed by Analysis Group, Inc. (*D. Macaulay, F. Mu, and E. Ohashi*) and interpreted in collaboration with all other authors (*T. Marshall, Y*. Jalundhwala, K. Sail, *and P*. Martinez‐Martin). Manuscript drafts were prepared by a professional medical writer ultimately paid by the sponsor, AbbVie, Inc., in collaboration with all authors. All authors take responsibility for the integrity of the data and the accuracy of the data analysis.

## Financial support

This study and manuscript were funded by AbbVie. The design, study conduct, and financial support for the study were provided by AbbVie. AbbVie participated in the study design, research, interpretation of data, writing, reviewing, and approving the manuscript.

## Full author disclosures (past 12 months)

T. Marshall, Y. Jalundhwala, and K. Sail are employees of AbbVie, Inc. and own stock/stock options. D. Macaulay, F. Mu, and E. Ohashi are employees of Analysis Group Inc., which has received consultancy fees from AbbVie, Inc. P. Martinez‐Martin has received honoraria from Editorial Viguera and AbbVie for lecturing, International Parkinson and Movement Disorder Society for management of the Program on Rating Scales, and HM Hospitales de Madrid for advice in a clinic‐epidemiological study; and license fee payments for the King's Parkinson's Disease Pain scale.

## Supporting information

Supplemental Table 1: Yearly Sample Size and Attrition Rates During the Five‐Year Study Period
Supplemental Table 2. Demographic Characteristics and Comorbidity Profile of PD Patients and Control Dependents during the Baseline Period^1^
Supplemental Table 3. Descriptive Mean Insurer Costs During the 5‐Year Study Period^1,2^
Supplemental Table 4. Descriptive Mean Out‐of‐Pocket Costs During the 5‐Year Study Period^1,2^
Supplemental Table 5. Descriptive Mean Indirect Costs During the 5‐Year Study Period^1,2^
