# Supplementary material for: The long‐term direct and indirect economic burden among Parkinson's disease caregivers in the United States
Source: Mov Disord. 2018 Dec 27;34(2):236–45. doi: 10.1002/mds.27579 (PMC6590233; doi:10.1002/mds.27579)
Supplement: Supplementary file 1 — Supplemental Table 1: Yearly Sample Size and Attrition Rates During the Five‐Year Study Period Supplemental Table 2. Demographic Characteristics and Comorbidity Profile of PD Patients and Control Dependents during the Baseline Period1 Supplemental Table 3. Descriptive Mean Insurer Costs During the 5‐Year Study Period1,2 Supplemental Table 4. Descriptive Mean Out‐of‐Pocket Costs During the 5‐Year Study Period1,2 Supplemental Table 5. Descriptive Mean Indirect Costs During the 5‐Year Study Period1,2 [file MDS-34-236-s001.docx]

**SUPPLEMENTAL MATERIAL**

**Supplemental Table 1: Yearly Sample Size and Attrition Rates During the Five-Year Study Period**

|  | **Year 1** | **Year 2** | **Year 3** | **Year 4** | **Year 5** |
| --- | --- | --- | --- | --- | --- |
| **Direct cost analysis, N (%)** |  |  |  |  |  |
| PD caregivers | 1,211 (100%) | 870 (72%) | 610 (50%) | 460 (38%) | 323 (27%) |
| Matched controls | 6,055 (100%) | 3,638 (60%) | 2,368 (39%) | 1,523 (25%) | 1,022 (17%) |
| **Indirect cost analysis, N (%)** |  |  |  |  |  |
| PD caregivers | 418 (100%) | 286 (68%) | 204 (49%) | 144 (34%) | 89 (21%) |
| Matched controls | 2,090 (100%) | 1,197 (57%) | 732 (35%) | 414 (20%) | 268 (13%) |
| **Income progression analysis, N (%)** |  |  |  |  |  |
| PD caregivers | 378 (100%) | 286 (76%) | 185 (49%) | 122 (32%) | 66 (17%) |
| Matched controls | 1,890 (100%) | 1,309 (69%) | 768 (41%) | 424 (22%) | 195 (10%) |

**Caption**

Abbreviations: N = number; PD = Parkinson's disease.

**Supplemental Table 2. Demographic Characteristics and Comorbidity Profile of PD Patients and Control Dependents during the Baseline Period^1^**

|  | **PD Patients** | **Control Dependents** | ***P*^2^** | |
| --- | --- | --- | --- | --- |
|  |  |  |  |  |
|  | N = 1,146 | N = 3,063 |  |  |
| **Demographic characteristics** |  |  |  |  |
| Sex, N (%) |  |  |  |  |
| Male | 621 (54.2%) | 1,134 (37.0%) | <0.01 | * |
| Age, years, mean (SD) | 58.57 (9.55) | 51.19 (11.38) | <0.01 | * |
| Region, N (%)^3^ |  |  |  |  |
| Northeast | 246 (21.5%) | 699 (22.8%) | 0.26 |  |
| Midwest | 280 (24.4%) | 728 (23.8%) |  |  |
| South | 439 (38.3%) | 1,149 (37.5%) |  |  |
| West | 181 (15.8%) | 487 (15.9%) |  |  |
| Insurance type, N (%) |  |  |  |  |
| Preferred provider organization | 677 (59.1%) | 1,732 (56.5%) | 0.16 |  |
| Point of service | 276 (24.1%) | 809 (26.4%) |  |  |
| Indemnity | 150 (13.1%) | 364 (11.9%) |  |  |
| Other | 43 (3.8%) | 158 (5.2%) |  |  |
| Relation to caregiver, N (%) |  |  |  |  |
| Spouse | 1,125 (98.2%) | 2,791 (91.1%) | <0.01 | * |
| Child | 15 (1.3%) | 257 (8.4%) |  |  |
| Handicapped | 4 (0.3%) | 3 (0.1%) |  |  |
| Sponsored | 1 (0.1%) | 6 (0.2%) |  |  |
| Class II dependent | 1 (0.1%) | 2 (0.1%) |  |  |
| Unknown | 0 (0.0%) | 4 (0.1%) |  |  |
| **CCI, mean (SD)^4^** | 0.74 (1.34) | 0.24 (0.74) | <0.01 | * |
| **Comorbidities included in the CCI, N (%)^5^** |  |  |  |  |
| Myocardial infarction | 17 (1.5%) | 9 (0.3%) | <0.01 | * |
| Congestive heart failure | 48 (4.2%) | 28 (0.9%) | <0.01 | * |
| Peripheral vascular disease | 31 (2.7%) | 18 (0.6%) | <0.01 | * |
| Cerebrovascular disease | 108 (9.4%) | 31 (1.0%) | <0.01 | * |
| Dementia | 28 (2.4%) | 1 (0.0%) | <0.01 | * |
| Chronic pulmonary disease | 79 (6.9%) | 105 (3.4%) | <0.01 | * |
| Rheumatologic disease | 30 (2.6%) | 29 (0.9%) | <0.01 | * |
| Peptic ulcer disease | 6 (0.5%) | 10 (0.3%) | 0.36 |  |
| Mild liver disease | 9 (0.8%) | 12 (0.4%) | 0.12 |  |
| Diabetes (mild to moderate) | 112 (9.8%) | 160 (5.2%) | <0.01 | * |
| Diabetes with chronic complications | 44 (3.8%) | 37 (1.2%) | <0.01 | * |
| Hemiplegia or paraplegia | 31 (2.7%) | 2 (0.1%) | <0.01 | * |
| Renal disease | 30 (2.6%) | 11 (0.4%) | <0.01 | * |
| Any malignancy, including lymphoma and leukemia | 56 (4.9%) | 86 (2.8%) | <0.01 | * |
| Moderate or severe liver disease | 1 (0.1%) | 1 (0.0%) | 0.49 |  |
| Metastatic solid tumor | 9 (0.8%) | 12 (0.4%) | 0.09 |  |
| HIV/AIDS | 0 (0.0%) | 0 (0.0%) | --^6^ |  |
|  |  |  |  |  |

**Caption**

Abbreviations: CCI = Charlson Comorbidity Index; HIV/AIDS = human immunodeficiency virus/acquired immunodeficiency syndrome; ICD-9= International Classification of Diseases, Ninth Revision; N = number; PD = Parkinson's disease; SD = standard deviation. **p*<0.05

Notes:

^1^ The baseline period was defined as the 6 months prior to the index date.

^2^ P-values were estimated by generalized estimating equations, which account for the correlation between the PD patients and control dependents due to the matching of PD caregivers and the non-PD controls.

^3^ The distribution of region is reflective of the regional distribution of claims data available and not the regional distribution of PD patients.

^4^ The 17 conditions included in the CCI were identified using ICD-9 diagnosis codes reported by Romano et al. [46] during the baseline period.

^5^ Comorbidities were defined using ICD-9 codes during the baseline period.

^6^ P-values were not available because no events were observed among PD patients or control dependents.

**Supplemental Table 3. Descriptive Mean Insurer Costs During the 5-Year Study Period^1,2^**

| **Insurer Costs, mean (SD)^3^** | **PD Caregivers** | **Matched Controls** | ***P*^4^** | |
| --- | --- | --- | --- | --- |
| **All-cause medical costs** |  |  |  |  |
| Year 1 | $5,902 ($17,006) | $5,940 ($24,328) | 0.95 |  |
| Year 2 | $5,420 ($19,997) | $5,083 ($18,131) | 0.65 |  |
| Year 3 | $5,665 ($19,137) | $5,188 ($16,125) | 0.56 |  |
| Year 4 | $5,151 ($12,598) | $5,715 ($20,198) | 0.48 |  |
| Year 5 | $5,484 ($13,925) | $4,992 ($16,329) | 0.57 |  |
| **Comorbidity-related medical costs^5^** |  |  |  |  |
| Year 1 | $1,548 ($5,362) | $1,291 ($5,606) | 0.11 |  |
| Year 2 | $1,501 ($5,513) | $1,323 ($6,521) | 0.40 |  |
| Year 3 | $1,416 ($4,756) | $1,361 ($5,807) | 0.81 |  |
| Year 4 | $1,534 ($7,469) | $1,462 ($7,354) | 0.85 |  |
| Year 5 | $1,283 ($5,242) | $1,144 ($4,148) | 0.70 |  |
| **Prescription drug costs** |  |  |  |  |
| Year 1 | $1,861 ($3,598) | $1,555 ($3,795) | <0.01 | * |
| Year 2 | $1,760 ($3,559) | $1,625 ($4,355) | 0.32 |  |
| Year 3 | $1,756 ($3,218) | $1,750 ($3,873) | 0.97 |  |
| Year 4 | $1,833 ($3,609) | $1,780 ($3,884) | 0.79 |  |
| Year 5 | $1,876 ($3,064) | $1,809 ($4,354) | 0.76 |  |
| **Total costs** |  |  |  |  |
| Year 1 | $7,763 ($17,917) | $7,495 ($25,329) | 0.66 |  |
| Year 2 | $7,180 ($20,641) | $6,708 ($19,546) | 0.54 |  |
| Year 3 | $7,421 ($19,847) | $6,938 ($17,260) | 0.57 |  |
| Year 4 | $6,984 ($14,153) | $7,495 ($21,264) | 0.55 |  |
| Year 5 | $7,360 ($15,141) | $6,801 ($17,522) | 0.56 |  |
|  |  |  |  |  |
|  |  |  |  |  |

**Caption**

Abbreviations: ICD-9 = International Classification of Diseases, Ninth Revision; PD = Parkinson's disease; SD = standard deviation. *p<0.05

Notes:

^1^ The study period for each patient was index date to the end of eligibility (maximum follow-up of 5 years) or until the patient was age 65.

^2^ Costs were calculated among all patients with eligibility during each year period.

^3^ Total insurer costs included costs included medical and drug costs and were calculated as the sum of paid amounts. Costs were adjusted for inflation to 2014 United States dollars using the Consumer Price index from the Bureau of Labor Statistics.

^4^ P-values were estimated by generalized linear models with Tweedie distribution and log link. The generalized estimating equations were used to account for the correlation between the PD caregivers and their matched controls.

^5^ Comorbidity-related medical costs were calculated as the sum of medical costs associated with an ICD-9 code for a comorbidity associated with caregiving.

**Supplemental Table 4. Descriptive Mean Out-of-Pocket Costs During the 5-Year Study Period^1,2^**

| **Out-of-Pocket Costs, mean (SD)^3^** | **PD Caregivers** | **Matched Controls** | ***P*^4^** | |
| --- | --- | --- | --- | --- |
|  |  |  |  |  |
| **Medical costs** |  |  |  |  |
| Year 1 | $812 ($2,070) | $708 ($1,271) | 0.07 |  |
| Year 2 | $813 ($1,295) | $699 ($1,062) | <0.01 | * |
| Year 3 | $789 ($1,047) | $754 ($1,251) | 0.46 |  |
| Year 4 | $863 ($1,181) | $740 ($1,305) | 0.04 | * |
| Year 5 | $894 ($1,303) | $690 ($1,021) | <0.01 | * |
| **Prescription drug costs** |  |  |  |  |
| Year 1 | $419 ($590) | $347 ($472) | <0.01 | * |
| Year 2 | $409 ($511) | $356 ($479) | <0.01 | * |
| Year 3 | $413 ($526) | $382 ($491) | 0.15 |  |
| Year 4 | $427 ($552) | $387 ($488) | 0.16 |  |
| Year 5 | $459 ($592) | $375 ($461) | 0.01 | * |
| **Total costs** |  |  |  |  |
| Year 1 | $1,231 ($2,224) | $1,055 ($1,465) | <0.01 | * |
| Year 2 | $1,222 ($1,525) | $1,055 ($1,275) | <0.01 | * |
| Year 3 | $1,202 ($1,303) | $1,136 ($1,450) | 0.24 |  |
| Year 4 | $1,290 ($1,471) | $1,128 ($1,526) | 0.03 | * |
| Year 5 | $1,354 ($1,552) | $1,065 ($1,253) | <0.01 | * |
|  |  |  |  |  |

**Caption**

Abbreviations: PD = Parkinson's disease; SD = standard deviation. *p<0.05

Notes:

^1^ The study period for each patient was index date to the earliest date among end of eligibility, end of continuous employment, or until the patient was age 65 (maximum follow-up of 5 years).

^2^ Costs were calculated among all patients with eligibility during each year period.

^3^ Total out-of-pocket costs included medical and drug costs and were calculated as the sum of deductible, coinsurance, and copayment amounts. Costs were adjusted for inflation to 2014 United States dollars using the Consumer Price Index from the Bureau of Labor Statistics.

^4^ P-values were estimated by generalized linear models with Tweedie distribution and log link. The generalized estimating equations were used to account for the correlation between the PD caregivers and their matched controls.

**Supplemental Table 5. Descriptive Mean Indirect Costs During the 5-Year Study Period^1,2^**

| **Indirect Costs, mean (SD)^3^** | **PD Caregivers** | **Matched Controls** | ***P*^4^** | |
| --- | --- | --- | --- | --- |
|  |  |  |  |  |
| **Medically related absenteeism costs^5^** |  |  |  |  |
| Year 1 | $1,550 ($2,499) | $1,207 ($2,660) | <0.01 | * |
| Year 2 | $1,511 ($3,169) | $1,212 ($2,207) | 0.10 |  |
| Year 3 | $1,612 ($3,159) | $1,370 ($3,208) | 0.32 |  |
| Year 4 | $1,137 ($1,268) | $1,299 ($2,628) | 0.29 |  |
| Year 5 | $1,304 ($1,693) | $1,163 ($1,570) | 0.48 |  |
| **Disability costs^6^** |  |  |  |  |
| Year 1 | $286 ($2,432) | $573 ($4,200) | 0.12 |  |
| Year 2 | $214 ($1,754) | $541 ($4,061) | 0.09 |  |
| Year 3 | $471 ($4,043) | $631 ($4,135) | 0.62 |  |
| Year 4 | $31 ($377) | $760 ($4,811) | <0.01 | * |
| Year 5 | $114 ($485) | $548 ($3,024) | <0.01 | * |
| **Total indirect costs^7^** |  |  |  |  |
| Year 1 | $1,836 ($3,396) | $1,781 ($4,926) | 0.78 |  |
| Year 2 | $1,725 ($3,605) | $1,753 ($4,547) | 0.93 |  |
| Year 3 | $2,083 ($5,074) | $2,001 ($5,124) | 0.84 |  |
| Year 4 | $1,168 ($1,319) | $2,058 ($5,461) | <0.01 | * |
| Year 5 | $1,417 ($1,753) | $1,711 ($3,321) | 0.28 |  |
|  |  |  |  |  |

**Caption**

Abbreviations: PD = Parkinson's disease; SD = standard deviation. *p<0.05

Notes:

^1^ The study period for each patient was index date to the earliest date among end of eligibility, end of continuous employment, or until the patient was age 65 (maximum follow-up of 5 years).

^2^ Costs were calculated among all patients with eligibility during each year period.

^3^ All costs were inflated to 2014 United States dollars using average hourly compensation data from the Bureau of Labor Statistics.

^4^ P-values were estimated by generalized linear models with Tweedie distribution and log link. The generalized estimating equations were used to account for the correlation between the PD caregivers and their matched controls.

^5^ Medically related absenteeism costs were calculated based on individual employee wage information obtained from eligibility files and days of medically related absenteeism. Medically related absenteeism days were imputed based on use of medical services during business days (e.g., an office visit or a hospital inpatient visit during Monday through Friday) as well as the waiting period in advance of the start of disability (e.g., 5 missed days of work due to illness). The methodology assumed that each hospitalization day and emergency department visit accounted for a full day of work loss, while each outpatient/other visit accounted for half a day of work loss.

^6^ Disability costs were based on actual employer disability payments.

^7^ Total indirect costs were calculated as the sum of disability and medically related absenteeism costs.
